# Bilateral Madelung Wrist Deformity in a 13-Year-Old Girl: Surgical Correction With the Taylor Spatial Frame External Fixation System

**DOI:** 10.5435/JAAOSGlobal-D-18-00036

**Published:** 2019-11-04

**Authors:** Panos Megremis, Orestis Megremis

**Affiliations:** From the A' Orthopaedic Department, Athens Children's Hospital, Athens, Greece.

## Abstract

Madelung deformity is a complex malformation of the wrist, due to growth disturbance in the volar and ulnar part of the distal radial physis. We report a bilateral idiopathic Madelung wrist deformity, in a 13-year-old girl, corrected surgically with the Taylor spatial frame external fixation system plus osteotomy. The Taylor spatial frame,a hexapod system of external fixation, has the ability, by distraction histogenesis, to simultaneously correct all components of this multiplanar three-dimensional wrist deformity, restoring gradually the distal radius morphology and radiocarpal alignment. Furthermore, the hexapod system, assisted with a web-based software program, allowed for, any time during the correction procedure, all proper modifications of the prescription needed, for the deformity correction. With this surgical technique, we achieved a full correction of the bilateral Madelung deformity and restored good function.

Madelung deformity of the wrist is a complex, multiplanar malformation of the wrist, due to growth disturbance in the volar and ulnar part of the distal radial physis. This growth disturbance results in carpal wedging, with the lunate at the apex of the wedge. In addition, this regional distal radial physis growth disturbance is responsible for the increased ulnar and palmar tilting of the distal radial articular surface, and for the formation of the bowed, shortened radial shaft, in conjunction with relative dorsal subluxation of the distal ulna. The most prominent clinical characteristics of this deformity are the volar translation of the hand and carpus at the distal radius and ulna joint and apex dorsal angulation of the distal radius at the metaphysis (Figure 1). It occurs predominantly in adolescent women, who are complaining of progressively increased wrist pain and volar translation of the hand and wrist and of progressively decreasing wrist motion, affecting mainly supination and dorsiflexion.

**Figure 1:**
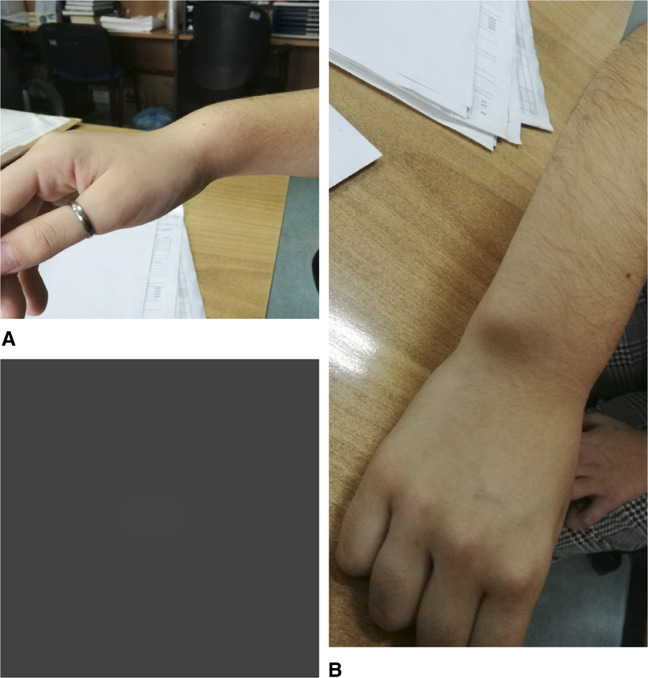
Photograph of Madelung deformity of the right wrist. **A**, Volar translation of the wrist and hand, relative to the longitudinal axis of the forearm. **B**, Dorsally prominent distal ulna.

Symptoms of Madelung deformity usually begin during adolescence, in girls aged 10 to 14 years.

In the skeletally immature patient, clear evidence of Madelung deformity has not been established that the most likely cause of pain is tension within the Vickers ligament.

Vickers ligament is a ligamentous structure, connecting the lunate bone and triangular fibrocartilage complex to the distal radius, and is seen in the vast majority of patients with Madelung deformity.

If the deformity has progressed in an older child, and the remaining growth is insufficient, a surgical procedure aiming to correct the malposition of the distal radiocarpal joint alignment can be used. The surgical procedure generally consists of a biplane osteotomy to the distal end of radius, in an attempt to correct the malposition of the distal radial articular surface and to restore a normal radiocarpal articulation. If a positive ulnar variance remains, and increased radial length with the radius osteotomy is insufficient, ulna-shortening procedure can be done.

Ulnar variance (also known as Hulten variance) refers to the relative lengths of the distal articular surfaces of the radius and ulna. The ulnar variance may be neutral (both the ulnar and radial articular surfaces are at the same level), positive (ulna projects more distally), or negative (ulna projects more proximally). In a positive ulnar variance, the ulnar and radial articular surfaces are not at the same level, and the distal articular surface of the ulna projects more distally than the articular surface of the radius. In such a case, a positive ulnar variance plays an important role in wrist pathology, such as ulnar impaction syndrome and thinning of the triangular fibrocartilage complex.

The use of a biplane osteotomy with immediate correction and fixation, in an attempt to correct this triplane deformity (distal end of the radius is in pronation, and increased palmar and ulnar tilting), requires surgical experience. In addition, there is always a risk of neurovascular damage, after the acute correction of this multiplanar wrist deformity.

A less commonly used method to correct Madelung deformity is distraction histogenesis, with the Ilizarov technique.^1,2,3,4,5^ Children treated with this method are either skeletally mature or nearly mature. Three or four rings are used. With the Ilizarov technique, several separate steps are needed to correct this multiplanar wrist deformity. In the first step, a period of over 3 weeks is usually needed, for the gradual correction of the triplane deformity of the distal end of the radius. In the next stage, an approximately 1.5-cm distal radius lengthening is necessary. A period of 2 weeks is usually needed for the radial lengthening, to bring both the ulnar and radial articular surfaces at the same level. Finally, a period of 4 to 6 weeks normally is required for the consolidation of the corrected and lengthened radius. The entire process to correct the wrist deformity takes over 9 weeks.

The Taylor spatial frame (TSF) system allows for the simultaneous correction of all components of a multiplanar limb malformation, such as the Madelung deformity, thus minimizing the time required for deformity correction.^6^ Furthermore, this hexapod system, assisted with a web-based software program, allows for all necessary modifications for deformity correction to be implemented at any time during the correction process. By contrast, the Ilizarov technique lacking the assistance of a web-based software program and the ability to simultaneously correct all the components of the wrist deformity demands more time for deformity correction and the availability of all the proper accessories to correct this complex, multiplanar wrist deformity.

To the best of our knowledge, only two reported post-traumatic, Madelung-like deformities, treated with the TSF external fixation system, are found.^6^

## Case Report

We report the correction of bilateral idiopathic Madelung deformity, in a 13-year-old girl, by callus distraction osteogenesis technique using the TSF external fixation system for the gradual restoration of the normal orientation of the radial articular surface and the simultaneous lengthening of the shortened radius.

This patient presented to our institution with distinctive the clinical features of Madelung deformity bilaterally with a volar translation of the carpus and hand and apex dorsal ulnar angulation of the distal radius (Figure 1). In addition, the dorsally prominent distal ulna was obvious, due to its relative dorsal subluxation (Figure 1), the estimated clinically 10° pronation of the distal end of the radius, and the restriction of wrist dorsiflexion and supination.

**Figure 2:**
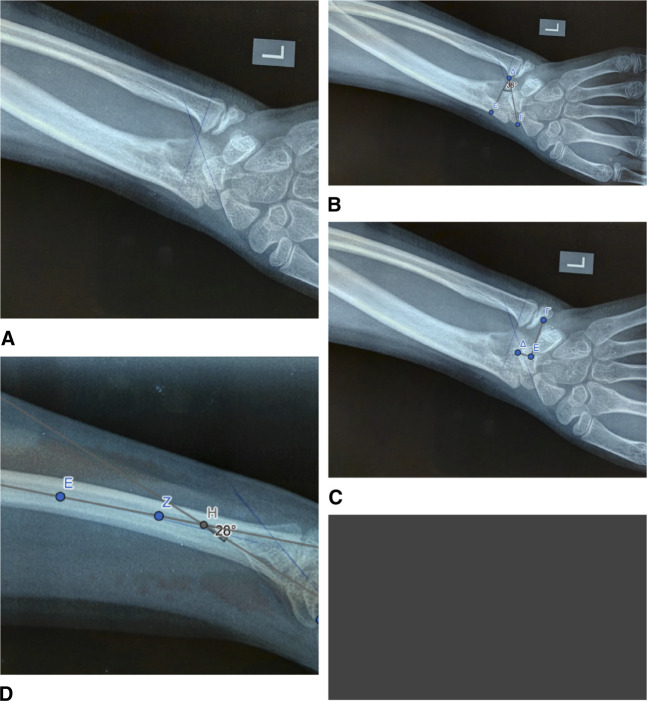
Radiographic findings of Madelung deformity of the left wrist. **A**, Frontal view. Focal osteopenia in the ulnar portion of the distal radius. Fusion of the ulnar half of the distal radial physis. Exostoses of the ulnar border of the distal radius. **B**, Frontal view. 38° ulnar tilting of the distal articular surface of the radius. **C**, Frontal view. Carpal wedging. 5-mm lunate proximal subsidence. **D**, Sagittal view. 28° volar tilting of the distal articular surface of the radius.

**Figure 3:**
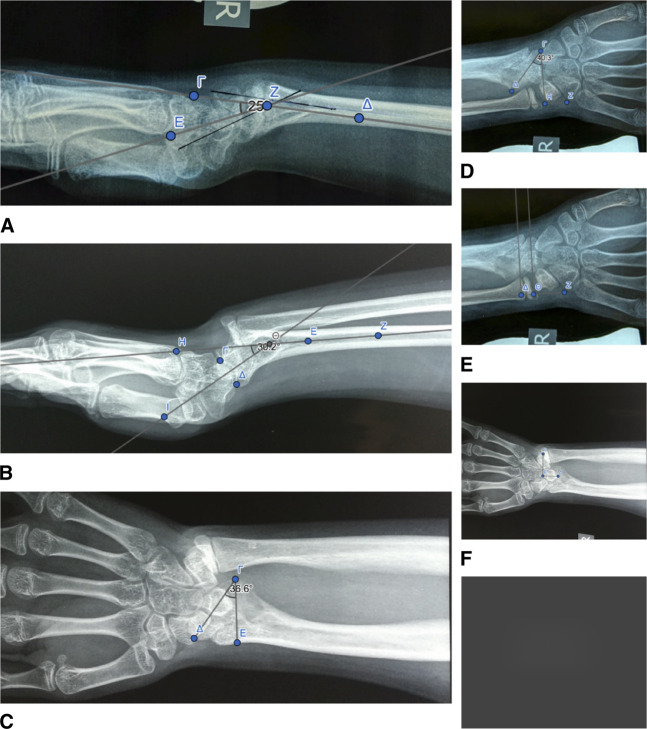
Madelung deformity of the right wrist. Radiographic findings in a 1-year time span. **A**, Sagittal view. 25° volar tilting of the distal articular surface of the right radius. **B**, Sagittal view. One year later, 30° volar tilting of the distal articular surface of the right radius. **C**, Frontal view. 36° ulnar tilting of the distal articular surface of the right radius. **D**, Frontal view. One year later, 40° ulnar tilting of the distal articular surface of the right radius. **E**, Frontal view. 5-mm lunate proximal subsidence of the right wrist. **F**, Frontal view. One year later, a 12-mm lunate proximal subsidence of the right wrist.

**Figure 4:**
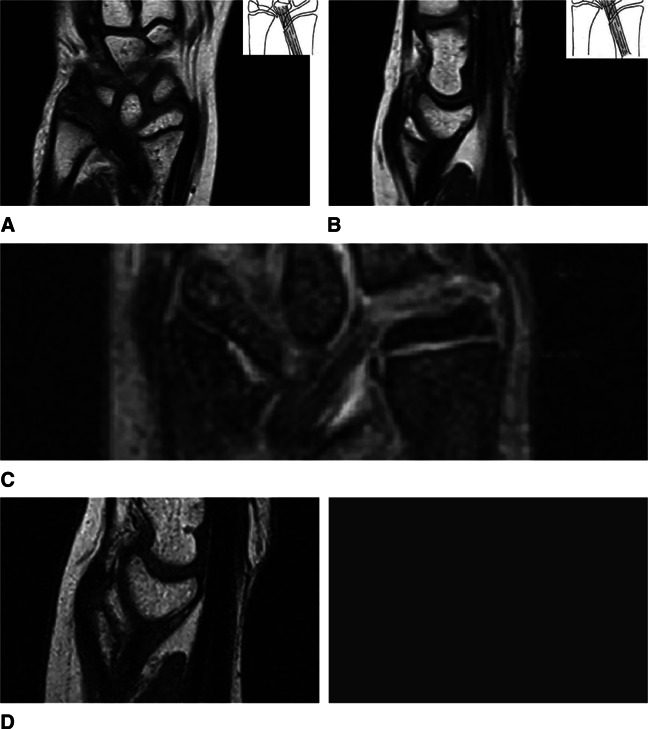
MRI of both wrists. Vickers ligament. **A**, Frontal plane. Vickers ligament of the left wrist. **B**, Sagittal plane. Vickers ligament of the left wrist. **C**, Frontal plane. Vickers ligament of the right wrist. **D**, Sagittal plane. Vickers ligament of the right wrist.

At the first clinical examination, there were distinctive radiological findings characteristic of a severe wrist deformity bilaterally, both on the P/A and lateral view (Figure 2). The P/A wrist view demonstrated characteristic features such as premature fusion of the ulnar half of the radial distal physis, focal osteopenia in the ulnar portion of the distal radius, indicative of the occupation of the ulnar corner of the radius by the fibrocartilaginous Vickers ligament, the exostoses of the ulnar border of the distal radius, and carpal wedging, forcing the lunate gradually to move proximally at the apex of the wedge (Figure 2). The lunate proximal subsidence (Figure 2) was found to be 5 mm in both wrists (lunate subsidence is the vertical distance between the most proximal point of the lunate and a line perpendicular to the longitudinal axis of the ulna, passing through its articular surface). Increased ulnar tilting of the distal articular surface of the radius was found. The distal articular surface of the radius had an ulnar tilting of 38° in the left wrist (Figure 2) and 36° in the right wrist (Figure 3), with a normal range of tilting (21° to 23°). On the lateral wrist image, an increased palmar tilting of the distal articular surface of the radius bilaterally was found. The distal articular surface of the radius had a palmar tilting of 28° in the left wrist (Figure 2) and 25° in the right wrist (Figure 3), with a normal range of palmar tilting (10° to 15°). On MRI of both wrists, the Vickers ligament was clearly seen (Figure 4). The calculated distraction lengthening of the distal radius needed for the restoration of a normal-oriented distal radial articular surface was 5 mm for both wrists that time. In addition, for the restoration of a normal-oriented distal radial articular surface in both wrists, the 10° pronation of the distal radius needed correction.

**Figure 5:**
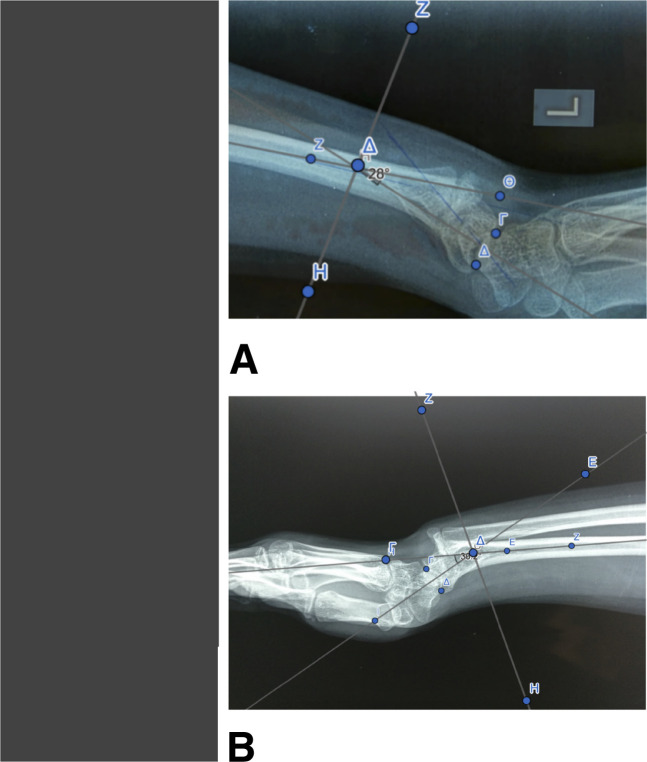
Distal radial osteotomy for Madelung deformity correction. **A**, Distal radial osteotomy of the left wrist. **B**, Distal radial osteotomy of the right wrist.

The surgical correction of the left wrist Madelung deformity was executed first because the left wrist Madelung deformity was clinically and radiologically worse than the right. The distal radial osteotomy was done as close as possible to the CORA deformity (Apex of the deformity), at the intersection of the proximal and distal anatomical axes of the radius. The ideal osteotomy was at the transverse bisector line (Figure 5). The radial osteotomy (1 cm proximal to the open growth insufficient physis) was combined with Vickers ligament release at the time of the procedure.

**Figure 6:**
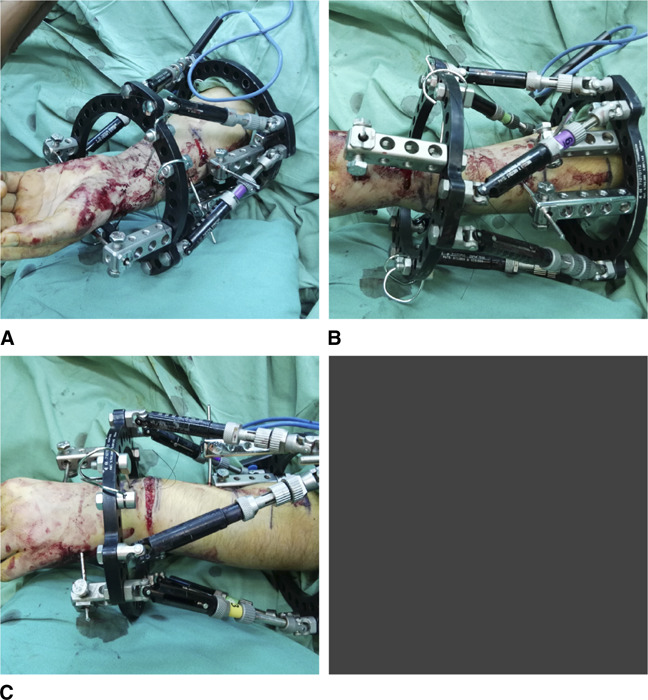
Photograph showing the intraoperative assemblage of the TSF system for the left Madelung deformity correction. **A**, The TSF system assembled with six fast FX struts and two 105-mm full rings, selecting the proximal ring as the reference ring. The reference ring placed orthogonal to the proximal radius. **B**, For the proximal ring fixation, two rancho cubes were used for the insertion of two 3.0-mm half pins to the proximal radius. **C**, For the distal ring fixation, two 1.5-mm smooth wires were inserted to the distal radius. For wrist stabilization, two 3.0-mm half pins were inserted to the base of the second and fifth metacarpal. TSF = Taylor spatial frame

**Figure 7:**
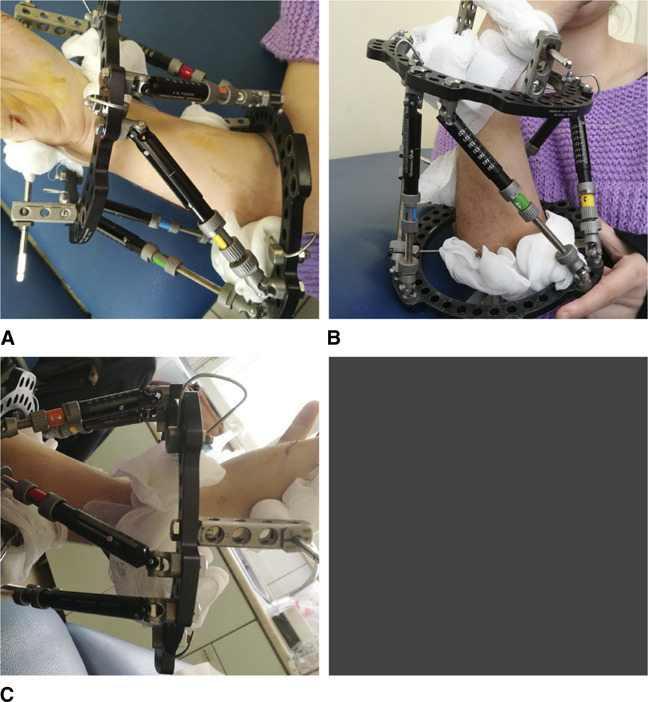
Photograph showing the assemblage of the TSF system for the right Madelung deformity correction. **A**, The TSF system assembled with six fast FX struts and two 130-mm full rings, selecting the distal ring as the reference ring. The reference ring placed orthogonal to the distal radius. For the proximal ring fixation, one rancho cube was used for the insertion of one 3.0-mm half pin to the proximal radius and one 1.5-mm smooth wire was used for the insertion to the proximal ulna. **B**, For wrist stabilization, two 3.0-mm half pins were inserted to the base of the second and fifth metacarpal. **C**, The distal ring was selected as the reference ring. The reference ring placed orthogonally to the distal radius. TSF = Taylor spatial frame

**Figure 8:**
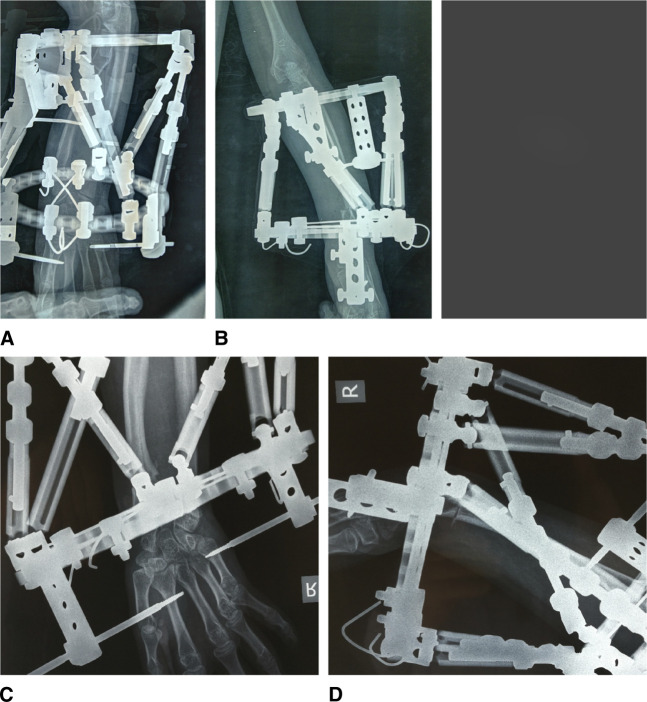
Radiographic view of the reference ring placed orthogonal to the reference fragment. **A**, Left wrist. A/P view. The proximal ring is the reference ring. **B**, Left wrist. Lateral view. The proximal ring is the reference ring. **C**, Right wrist. A/P view. The distal ring is the reference ring. **D**, Right wrist. Lateral view. The distal ring is the reference ring.

At 1-year follow-up, the left wrist demonstrated good alignment of the forearm/hand relationship, with increased supination and dorsiflexion (Table 1). The 1-year follow-up also showed worsening of the right wrist Madelung deformity, with its distal articular surface volar and ulnar tilting increased to 30° and 40°, respectively (Figure 3). In addition, the lunate proximal subsidence was increased to 12 mm (Figure 3). The calculated distraction lengthening of the distal radius needed for the restoration of a normal-oriented right distal radial articular surface was 12 mm. The surgical correction of the right wrist Madelung deformity with the use of the TSF was executed then. Complex limb deformity correction with external unilateral or circular Ilizarov ring fixators, using the callus distraction osteogenesis technique, is used in children but requires frequent, time-consuming frame modifications. The TSF system, allowing for the progressive, simultaneous correction of all the components of a multiplanar malformation of a limb, such as the Madelung deformity of the wrist, minimizes the required time of deformity correction. Furthermore, this hexapod system, assisted with a web-based software program, allowed the proper modifications of the prescription needed and more precise correction of the deformity. The TSF system was assembled with six fast FX struts and with two 105-mm full rings for the left wrist (Figure 6) and with two 130-mm full rings for the right wrist (Figure 7), selecting the proximal ring as the reference ring for the left wrist (Figures 6 and 8) and the distal ring as the reference ring for the right one (Figures 7 and 8). The reference ring was placed orthogonal to the reference fragment (proximal or distal radius). For the distal ring fixation, two 1.5-mm smooth wires were inserted to the distal radius (Figures 6 and 7). For the proximal ring fixation, two rancho cubes (Figure 6) were used for the insertion of two 3.0-mm half-pins to the proximal left radius. On the contrary, one rancho cube was used for the insertion of one 3.0-mm half pin to the proximal right radius and one 1.5-mm smooth wire was inserted to the proximal right ulna (Figure 7). The incorporation to the distal ring, bilaterally, of two 3.0-mm half pins, inserted to the second and fifth metacarpal, was necessary to stabilize the wrist joint and to prevent its collapse (Figures 6 and 7).

**Table 1:** Madelung Deformity: Preoperative and Postoperative Data ROM

Factor	Deformity Preoperative						Postop. ROM			Frame		Deformity Postoperative						Postop. ROM.		
	S, mm	AU	AV	TV mm	LUN SUB, mm	R.PR	FL/EXT	PR/SUP	RAD/ULN	Distraction, d	In Frame, d	S	A.U.	A.V.	T.V.	LUN. SUB	R.PR	FL/EXT	PR/SUP	RAD/ULN
Left wrist	5	38°	28°	10	5	10°	75°/40°	85°/40°	5°/35°	26	56	0	20°	12°	0	0	0	75/70°	85/80°	20/35°
Right wrist	12	40°	30°	10	12	10°	75°/40°	85°/40°	5°/35°	28	58	0	20°	11°	0	0	0	75/70°	85/80°	20/35°

AU = angulation ulnar; AV = angulation volar; FL/EXT = flexion/extension; LUN SUB = lunate subsidence; PR/SUP = pronation/supination; R.PR = rotation, pronation; RAD/ULN = radial/ulnar deviation; S = shortening; TV = translation volar

**Figure 9:**
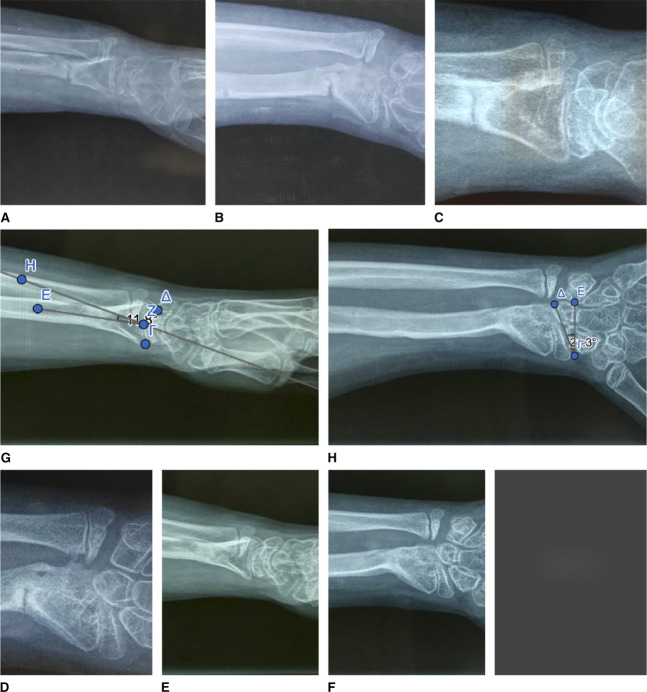
Radiographic view showing restoration of a normal left radiocarpal articulation. **A**, Lateral view. Anatomically aligned forearm/hand relation of the left wrist, immediately after TSF system removal. **B**, A/P view. Restoration of a normal left radiocarpal articulation, immediately after TSF system removal. **C**, Lateral view, immediately after cast removal. **D**, A/P view, immediately after cast removal. **E**, Lateral view, four months after TSF removal. **F**, A/P view, four months after TSF removal. **G**, Lateral view. 11.8° volar tilting of the distal articular surface of the radius. **H**, Frontal view. 20° ulnar tilting of the distal articular surface of the radius. TSF = Taylor spatial frame

**Figure 10:**
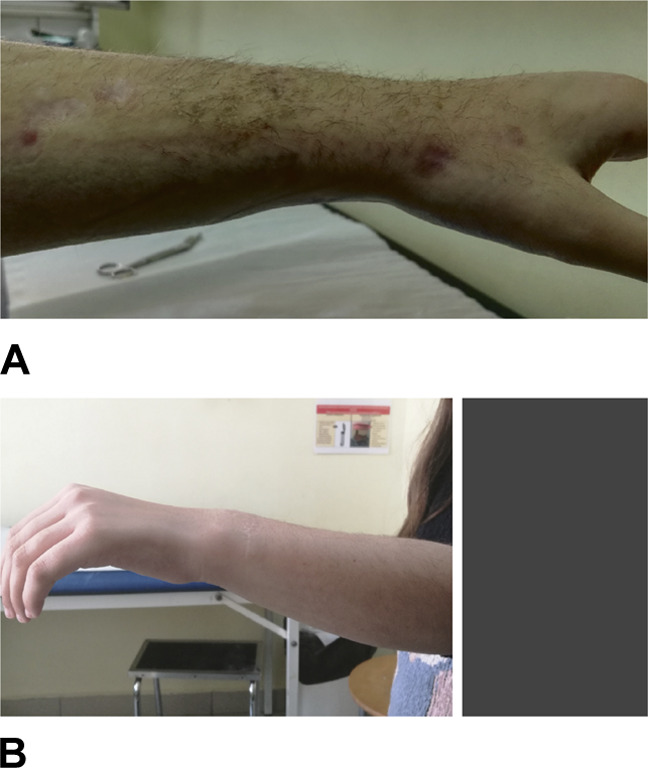
Photograph of the left wrist. Restoration of a normal left radiocarpal articulation. **A**, Immediately after cast removal. **B**, Two years postoperatively.

Precise A/P and lateral images are absolutely necessary to define the six deformity parameters (Figure 8). The web software gave us the calculated minimum correction time of 26 days for the left wrist and 28 for the right wrist, and the schedule the patient was expected to follow for struts adjustments every day. This calculation was based on the six deformity measurements, the four settings of the reference ring, the initial six struts settings, the defined structures at risk, and the entered maximum safe distraction rate of 0.5 mm/d. The initiation of correction was started at the seventh postoperative day. The multiplanar deformity of the distal radius was corrected anatomically at the end of the scheduled prescription. The web-based planning program was adjusted twice until total deformity correction was achieved. The frame was removed, when callus formation was achieved (Figure 9). A further immobilization of the wrist with a fiberglass cast for 3 weeks was required (Figure 9) after frame removal. The 1-year follow-up for the right wrist and the 2-year follow-up for the left wrist showed a good aligned forearm/hand relation (Figure 10) with increased wrist supination, radial deviation, and dorsiflexion (Table 1), compared with the preoperative range of motion.

## Discussion

Complex limb deformity correction with external unilateral or circular Ilizarov ring fixators, using the callus distraction osteogenesis technique, is used in children but requires frequent, time-consuming frame modifications. The TSF system, allowing for the simultaneous correction of all the components of a multiplanar malformation of a limb, such as the Madelung deformity of the wrist, minimizes the required time of deformity correction. Furthermore, this hexapod system, assisted with a web-based software program, allowed the proper modifications of the prescription needed and more precise correction of the deformity.

## Conclusion

The TSF system of external fixation system permits gradual correction of the rotational and translational components of the multiplanar Madelung wrist deformity without any need for frame modifications, which are necessary with the standard Ilizarov system.
